# Fatal Disseminated Aspergillosis in a Patient with Systemic Lupus Erythematosus

**DOI:** 10.1155/2020/9623198

**Published:** 2020-02-29

**Authors:** Rochelle Hardie, Tracian James-Goulbourne, Monsoon Rashid, Jeremy Sullivan, Yamen Homsi

**Affiliations:** ^1^Division of Infection Disease, SUNY Downstate Medical Center, 450 Clarkson Avenue, Brooklyn, NY 11203, USA; ^2^Division of Internal Medicine, SUNY Downstate Medical Center, 450 Clarkson Avenue, Brooklyn, NY 11203, USA; ^3^Division of Internal Medicine, NYU Langone Hospital-Brooklyn, 150 55th St, Brooklyn, NY 11220, USA; ^4^Division of Rheumatology, NYU Langone Hospital-Brooklyn, 150 55th St, Brooklyn, NY 11220, USA

## Abstract

Patients with systemic lupus erythematosus (SLE) are at increased risk for infection including opportunistic infections. Fungal infection in particular can be difficult to diagnose and treat and often can be life-threatening in the immunocompromised patient. We present a case in which a patient with SLE presented to the hospital with shortness of breath and cough. Throughout the hospital course, the patient's condition continued to decline leading to acute respiratory failure, and eventually, the patient expired. Postmortem autopsy revealed invasive fungal aspergillosis infection involving the heart, lungs, and brain. Earlier diagnosis and treatment with empiric antifungals may improve survival in these patients.

## 1. Introduction

Infection is a main cause of morbidity and mortality in patients with SLE [1]. Pneumonia, urinary tract, and skin infections are common in SLE patients; however, these patients are also at increased risk for opportunistic infections [2]. Immunosuppressive treatment also increases the risk of opportunistic infections [2]. Without early diagnosis and treatment, infection can rapidly progress and lead to death. In addition, infections in an SLE patient often mimic an SLE flare and make it harder to recognize the underlying infection. We report a 41-year-old female with SLE who presented with shortness of breath and cough. She expired quickly, and autopsy revealed a disseminated aspergillosis.

## 2. Case Presentation

A 41-year-old Guyanese woman presented to the emergency department with a chief complaint of progressively worsening shortness of breath, associated with productive cough of blood-tinged yellow sputum for three days. Over the same period, she reported subjective fevers, arthralgia, and generalized weakness. She denied any chills, chest pain, abdominal pain, and changes in bowel habits or urinary symptoms. She did not have a history of recent travel, tick bites, or ill contacts. Her past medical history was remarkable only for systemic lupus erythematosus (SLE), diagnosed three years before. She developed class IV lupus nephritis, which was diagnosed four months prior to her presentation. Following diagnosis of lupus nephritis, she was treated with three days of pulse methylprednisolone and one cycle of IV cyclophosphamide and then switched to mycophenolate mofetil (MMF). Her medications at presentation included MMF 500 mg three times daily, prednisone 20 mg daily, and hydroxychloroquine 400 mg daily. She never smoked, consumed alcohol, or used illicit drugs.

**Figure 1:**
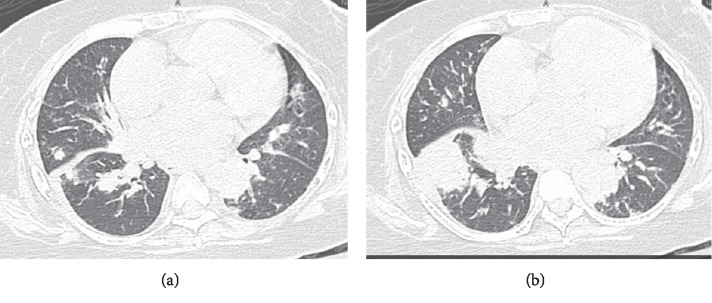
CT chest without contrast showed multifocal consolidations bilaterally with bibasilar predominance.

In the ED, vital signs were as follows: temperature 98.9 F, blood pressure 110/60 mmHg, heart rate 90 bpm, and respiratory rate 22 breaths per minute with oxygen saturation of 96% on room air. She was alert and oriented. Head, eye, ear, neck, and throat examination was significant for oral candidiasis. She had no lymphadenopathy. Cardiovascular examination showed normal S1 and S2 and no murmurs or gallop. Crackles were heard over the lower lung fields. No abnormalities were noted on the abdominal examination. Chest X-ray showed bilateral multifocal patchy opacities. CT chest without contrast showed multifocal consolidations bilaterally with bibasilar predominance concerning for multifocal pneumonia (Figure 1). She was placed on broad spectrum antibiotics with vancomycin and piperacillin/tazobactam, and she was continued on prednisone 20 mg/daily. Laboratory testing on admission and four months prior are summarized in (Table 1). Echocardiogram showed decreased ejection fraction to 25% (baseline 50–55%), which was attributed to demand ischemia in setting of severe sepsis. Within a week of admission, the patient developed hemoptysis and became hypoxic with altered mental status requiring intubation. Noncontrast head CT was negative for any acute intracranial event.

**Figure 2:**
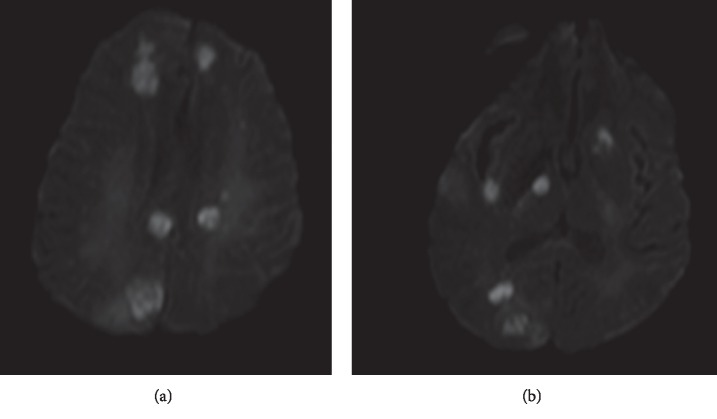
Image of MRI brain showing multifocal areas of diffusion restriction suggestive of vasculitis versus acute infarcts.

Given the concern for SLE flare, she received three days of pulse methylprednisolone, and plasma exchange was started for possible pulmonary hemorrhage related to SLE. Subsequent laboratory tests showed worsening renal function, uptrending white blood cells (up to 34.05 K/uL on admission day 5), and downtrending platelets (17 K/uL on admission day 6). Her kidney function declined, and she required hemodialysis. Gram-negative coverage was upgraded to meropenem. Culture of CSF, blood, urine, and sputum was unremarkable. Lumber puncture was performed on day 13 of admission. CSF fluid analysis is summarized in (Table 2). Empiric antifungal treatment was initiated with caspofungin. On day 13 of admission, she underwent bronchoscopy and bronchoalveolar lavage (BAL). *Candida glabrata* was isolated from the fungal cultures. Aspergillus was not isolated; however, serum Galactomannan testing was positive. Acid-Fast Bacilli (AFB) was also negative. Serum cryptococcal antigen was negative. She continued to spike temperatures up to 104 F and on admission day 14, had seizure-like activity with possible right-sided paresis. MRI brain without contrast was significant for multifocal areas of diffusion restriction, of which several had corresponding signal dropout that was thought to be secondary to vasculitis versus acute infarcts (Figure 2). Further evaluation with a transesophageal echocardiogram was significant for a possible hypodensity on the mitral valve. On day 20, the patient suffered a cardiac arrest with unsuccessful attempts at resuscitation. Autopsy was performed with consent from her family.

**Figure 3:**
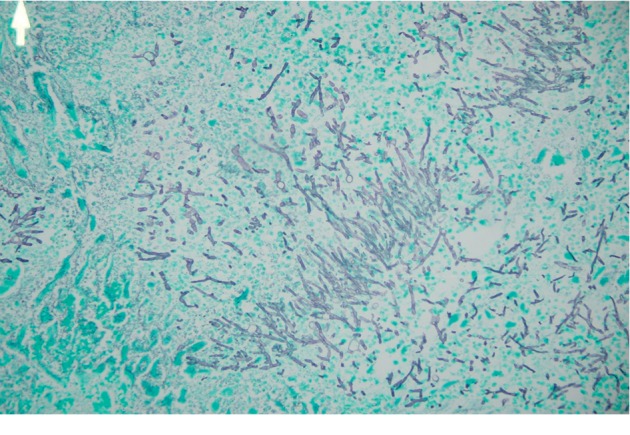
Histopathological slide showing a section from the Heart.

**Figure 4:**
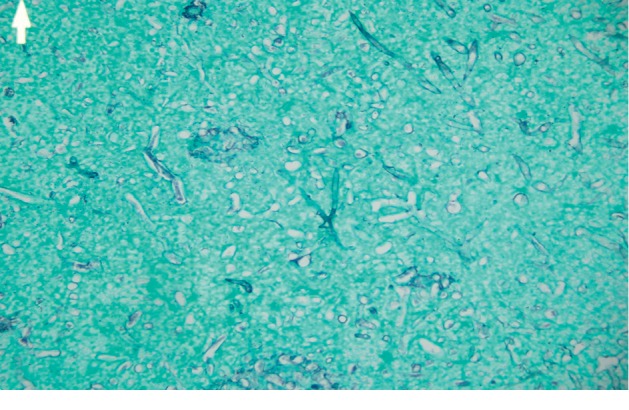
Histopathological slide showing a section from the Lung.

**Figure 5:**
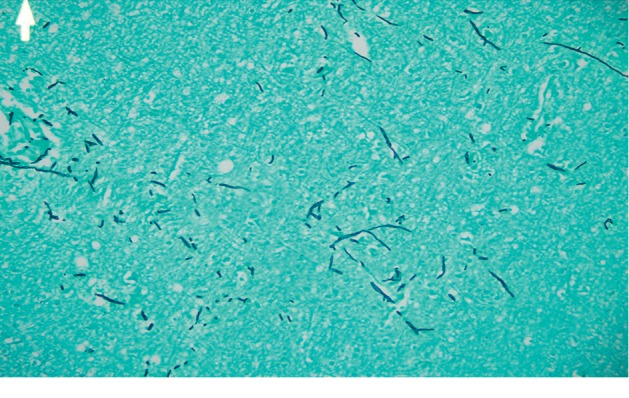
Histopathological slide showing a section from the Brain.

Autopsy revealed severe fungal infection in the myocardium, lungs, and brain (Figures 3–5). The neuropathology examination showed vascular necrosis with acute inflammation throughout the cerebral cortex, subcortical gray and white matters, brainstem, cerebellum, and spinal cortex. Many of the vascular lesions contained fungal hyphae consistent with aspergillosis. Large angiocentric necrosis in the cerebral cortex, pons, cerebellar cortex, and spinal cord were notable for fungal hyphae. The severity of this infection was noted to be incompatible with life.

## 3. Discussion

Systemic lupus erythematosus (SLE) is multisystem autoimmune disorder with a broad spectrum of clinical presentations encompassing almost all organs and tissues. Infection is a serious complication and principal cause of morbidity and mortality in patients with SLE [1]. In a large multicenter European SLE cohort of 100 patients followed over 10 years, infections represented the cause of death in a quarter of the cases [1]. Another US national population-based cohort study that compared the rate of serious infection in SLE patients to the general population showed a substantial increase of in-hospitalization rates of infection, as well as higher in-hospital mortality and opportunistic infection among SLE patients, when compared to patients without SLE [2]. Pneumonia, urinary tract, and skin infection are among the most reported infections causing hospitalization in SLE; subsequently, bacteremia and sepsis complicated with organ failure are the major causes of in-hospital mortality [2]. In the same cohort, among opportunistic infections, mortality was highest for listeriosis (27%), followed by pneumocystis (14%), aspergillosis (13%), nontuberculous mycobacterial infection (11%), candidiasis (11%), and cytomegalovirus (10%) [2]. Many studies investigated fungal infections in SLE patients. A retrospective study performed in southern Taiwan identified twenty SLE patients with invasive fungal infection (IFI) [3]. Among them, eight cases were disseminated, six involved the central nervous system, four involved the lungs, one involved abdomen, and one involved soft tissue. IFI attributed to 50% mortality among identified patients and was proved postmortem in three patients. The survival period from the diagnosis of IFI to death was only 7.7 ± 4.2 days [3]. Cryptococcus was the most common identified fungus in this series (70%); however, invasive Aspergillus of the lung was identified in one case. Other types of fungi included *Candida albicans* and *Penicillium marneffei* [3]. A similar study was conducted in China and investigated hospitalized SLE patients with IFI, and lung and CNS were the most common IFI sites accounting for more than 80% of SLE-IFI cases [4]. Cryptococcus, Aspergillus, and Candida were the most frequent pathogens among approximately 80% of cases [4]. Another retrospective multicenter cohort study evaluated IFI in patients with childhood-onset systemic lupus erythematosus (cSLE). Twenty-two cases were proven IFI, and six of them were Aspergillosis involving the lungs, CNS, and myocardium. The most important types of IFI leading to death in these series were eight cases of aspergillosis and six cases of candidiasis [5]. Some researchers focused specifically on evaluating invasive aspergillosis (IA) in SLE patients. Twenty-one SLE patients with IA were identified by reviewing medical records in a tertiary hospital in Taiwan, and review showed that 14 patients died [6]. The most common organ involvement with IA was the lungs and CNS. Risk factors for IA were investigated in this study. High daily steroid dose more or equal to 20 mg of prednisone, recent pulse steroid therapy, use of immunosuppressant like azathioprine or rituximab or concurrent infection, and CMV viremia were mortality risk factor for IA in SLE patients [6]. A case-control study analyzed associated factors for development of IFA in SLE patients [7]. Statistically significant associated factors were high C-reactive protein (CRP > 10 mg/dl with sensitivity of 74% and specificity of 70 for diagnosis of IFI), high dose prednisone (>0.5 mg/kg/day), low CH50, requirement of mechanical ventilation and hemodialysis, and treatment with antibiotics or use of mycophenolate mofetil [7]. In the same study, IFI contributed to 7 deaths, and only 7 out of 10 patients received antifungal therapy. Three patients did not receive any specific treatment because their diagnosis was made postmortem. Galactomannans and D-glucan are two the biomarkers which are used widely in diagnosis of IP. In one study, the sensitivity and the specificity of the serum GM were 82% and 81%, respectively, to detect IPA in neutropenic patients; however, in nonneutropenic patients, serum GM assays result in significantly poorer results [8]. In another retrospective study evaluating critically ill patients, serum GM was increased in only 53% of patients with IPA [9]. Finally, to determine early management and establish probable cause of acute respiratory failure in immunocompromised adults, critical diagnostic investigations are needed to be applied to such patients. Azouly and colleagues summarized a standardized initial and basic diagnostic that should be performed immediately at the admission time for all immunocompromised patients with acute respiratory failure [10].

Our patient received pulse steroids and continued high-dose prednisone afterward, in addition to receiving cyclophosphamide then MMF. This all attributed to the disseminated aspergillosis. In general, infection in SLE patients is a challenging diagnosis since it mimics SLE flare, and many patients ended up with more immunosuppressant treatment which plays a significant role in disseminating the underlying infection. Fungal infection carries a unique challenge since it is not initially suspected or recognized as a possible pathogen in infected SLE patients. This case reinforces the importance of early recognition fungal infection, prompt treatment with antifungal medication, and avoidance of immunosuppressive agents if not indicated, to improve survival in such patients.

Figures 3–5 show histopathological slides showing sections from the heart, lung, and brain with significant infiltration of aspergillus fumigatus hyphae by Grocott's methenamine silver stain.

## Figures and Tables

**Table 1:** Blood and urine studies.

Complete blood count	Current visit	Four months prior	Reference range
White blood cells	11.07	4.75	(4.5–10.9) K/uL
Neutrophils	84.0	59.7	(38.7–60.3) %
Bands	12.0	0	(0.0–10.0) %
Lymphocytes	2.0	26.5	(22.4–49.0) %
Monocytes	2.0	8.7	(2.4–9.2) %
Eosinophils	0	1.3	(0–8.6) %
Basophils	0	1.3	(0–1.0) %
Hgb	6.7	8.1	(12.0–16.0) g/dL
Hct	22.3	26.6	(37.0–47.0) %
RDW	19.8	19.4	(11.5–15.1) %
Platelets	111	210	(130–400) K/uL
Metabolic panel			
Sodium	139	141	(136–141) mmol/L
Potassium	4.7	4.1	(3.5–5) mmol/L
Chloride	99	105	(98–106) mmol/L
CO_2_	21	20	(24–31) mmol/L
Plasma urea nitrogen (BUN)	70	43	(6–20) mg/dL
Creatinine	2.88	2.35	(0.4–1.2) mg/dL
Glucose	117	79	(70–99) mg/dL
Calcium	8.2	8.6	(8.4–10.3) mg/dL
Total protein	4.6	6.2	(6.0–8.5) g/dL
Albumin	2.5	3.2	(2.8–5.7) g/dL
Aspartate amino transferase	20	28	(10–35) u/l
Alanine aminotransferase	29	23	(0–31) u/l
Alkaline phosphatase	143	69	(25–125) u/l
Total bilirubin	1.49	0.39	(0.0–1.2) mg/dL
LDH	222	n/a	(140–280) u/l
Rheumatology workup:			
ANA	1 : 640 homogenous pattern	1 : 640 speckled pattern	<1 : 40
Anti-SSA	Negative	Negative	Negative
Anti-SSB	Negative	Negative	Negative
Anti-Smith	Negative	Negative	Negative
Anti-ds DNA	130	65	(<100) U/mL
Rheumatoid factor	<7	<7	(<14) IU/mL
Anticyclic citrullinated peptide antibodies	10	10	(0–19) units
Anti-RNP	Negative	Negative	Negative
Creatine Kinase	32	30	(24–195) u/l
ESR	76	38	(0–20) mm/hr
hsCRP	>300	38.59	(1.0–4.0) mg/L
C3	87	45	(86–184) mg/dL
C4	26	22	(20–58) mg/dL
Total complement CH50	52	50	(42–60) U/mL
c-ANCA (PR3)	5	8	AU/mL <100—neg 100–120—equivocal >100—pos
p-ANCA (MPO)	5	10	AU/mL <100—neg 100–120—equivocal >100—pos
Others blood tests:			
Hep B Surface antibody	<3.5	<3.5	<8.499 mIU/mL
Hepatitis B Surface Ag	Nonreactive	Nonreactive	Nonreactive
Hep C antibody	Nonreactive	Nonreactive	Nonreactive
Quantiferon	Negative	Negative	Negative
Pro-BNP	>7000	110	(</ = 125) pg/ml
Urine protein/creatinine ratio	6.3	3.7	0.5 mg/g

**Table 2:** CSF analysis.

Variable	Result	Reference range
CSF appearance	Clear-colorless	Clear-colorless
CSF WBC	1	0–5 ul
CSF RBC	6–10	0 ul
CSF glucose	60	40–70 mg/dl
CSF total protein	95	15–45 mg/dl
CSF cryptococcal antigen	Negative	Negative
CSF CMV PCR	Negative	Negative
CSF EBV PCR	Negative	Negative
CSF HSV PCR	Negative	Negative
CSF culture	No growth after 72 hrs	No growth
